# Infective endocarditis on a patent ductus arteriosus revealed by a ruptured pulmonary artery trunk aneurysm in the pericardium: a case report

**DOI:** 10.1093/ehjcr/ytaf267

**Published:** 2025-05-24

**Authors:** Meryem Haboub, Mohamed Khaldi, Abdenasser Drighil

**Affiliations:** Cardiology Department, Hospital University Ibn Rochd, 8, Street Lahcen El Arjoun, Casablanca 20100, Morocco; Cardiology Department, Hospital University Ibn Rochd, 8, Street Lahcen El Arjoun, Casablanca 20100, Morocco; Cardiology Department, Hospital University Ibn Rochd, 8, Street Lahcen El Arjoun, Casablanca 20100, Morocco

**Keywords:** Case report, Patent ductus arteriosus, Infective endocarditis, Pulmonary artery aneurysm, *Staphylococcus aureus*

## Abstract

**Background:**

Infective endocarditis on a patent ductus arteriosus (PDA) is a rare but serious condition that can lead to life-threatening complications, such as mycotic aneurysms. We report a case of endocarditis on a PDA, complicated by multiple mycotic aneurysms, including a partially ruptured aneurysm of the pulmonary artery (PA) trunk.

**Case summary:**

A 20-year-old female presented with progressive dyspnoea, worsening over 48 h, accompanied by fever. Echocardiography revealed a large pericardial effusion with cardiac tamponade, necessitating the drainage of 500 mL of haemorrhagic fluid. Imaging studies revealed a false aneurysm of the PA trunk, with contrast extravasation into the pericardial space. Blood cultures and pericardial fluid cultures were positive for *Staphylococcus aureus*. Emergency surgery involved resection of the aneurysm, ligation of the PDA, and pericardial drainage. The patient recovered after 4 weeks of intravenous antibiotics and was discharged with good clinical and biological outcomes.

**Conclusion:**

This case illustrates the importance of early diagnosis and management of infective endocarditis associated with congenital heart defects, as the delay can result in severe complications such as aneurysm rupture.

**Learning points**

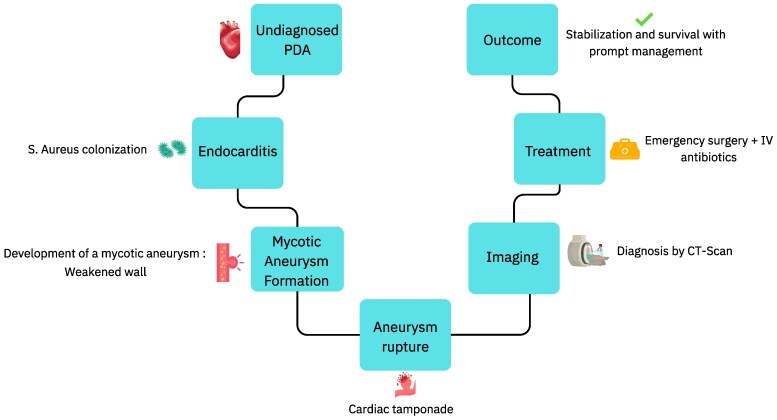

- *Staphylococcus aureus* infective endocarditis of a patent ductus arteriosus can be complicated of pulmonary artery mycotic aneurysms: a rare but serious complication.
- Pulmonary artery mycotic aneurysm can rupture in the pericardium and lead to haemorrhagic cardiac tamponade.

## Introduction

Infective endocarditis (IE) is a severe condition that can occur in patients with patent ductus arteriosus (PDA).^1^

Although rare, IE involving a PDA can lead to life-threatening complications, such as mycotic aneurysms.^2^ Mycotic aneurysms are dilations of arterial walls caused by infection and can rupture,^3^ resulting in significant morbidity and mortality.^4^

To our knowledge, the severe presentation with acute rupture of a pulmonary artery (PA) trunk mycotic aneurysm and cardiac tamponade has never been reported in the literature.

We are reporting a unique case of IE on a PDA, complicated by multiple mycotic aneurysms, including a partially ruptured aneurysm of the PA trunk in the pericardium.

## Summary figure

**Figure:**
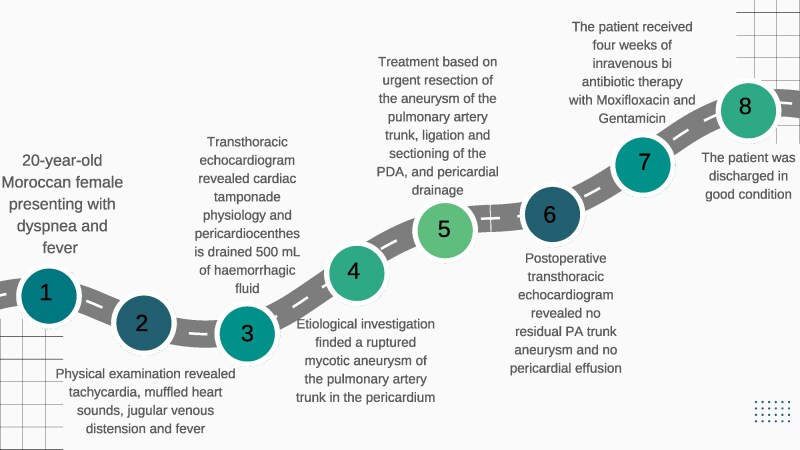


## Case presentation

A 20-year-old female with no known medical history presented to the emergency department with 1 month of progressive dyspnoea. The symptoms had worsened over the past 48 h and were associated with fever, fatigue, and reduced exercise tolerance.

On examination, the heart rate was of 155 b.p.m., the blood pressure of 110/70 mmHg, the temperature of 39.5°C, and the oxygen saturation of 87% on room air. Jugular venous distension was observed, and heart sounds were muffled. There was no peripheral oedema, and the lungs were clear on auscultation.

The electrocardiogram revealed sinus tachycardia at 155 b.p.m., low QRS voltage, and electrical alternans.

Transthoracic echocardiogram (TTE) showed a large circumferential pericardial effusion, with signs of right ventricular collapse and significant respiratory variation in flow, consistent with cardiac tamponade physiology. The inferior vena cava was dilated. Immediate pericardiocenthesis was performed, and 500 mL of haemorrhagic fluid was drained.

Blood tests reveal 7.3 g/dL hypochromic and microcytic anaemia (normal range of haemoglobin: 13–17 g/dL), 17 690/mm^3^ leukocytosis (normal range: 4000–10 000/mm³), neutrophils 15 740/mm^3^ (normal range: 2000–7500/mm³), C-reactive protein level of 247 mg/L (normal range: <5 mg/L), procalcitonin level of 32 ng/mL (normal range: <0.5 ng/mL), thrombopenia platelets count of 100 000/mm³ (normal range: 150 000–400 000/mm³), blood urea nitrogen of 2.22 g/L (normal range: 0.15–0.42 g/L), serum creatinine of 31 mg/L (normal range: 6–12 mg/L) eGFR of 20 mL/min/1.73 m², PT of 70% (normal range: 70–140%), APTT of 29 s, fibrinogen of 6 g/L (normal range: 2–6 g/L), serum potassium level of 4.1 mEq/L (normal range: 3.5–4.5 mEq/L), serum sodium level of 137 mEq/L (normal range: 135–145 mEq/L), and a positive direct Coombs test. Blood cultures were positive for methicillin-susceptible *Staphylococcus aureus*.

Pericardial fluid analysis revealed haemorrhagic fluid with 7250 cells/µL, 98% neutrophils, negative for malignant cells, and tuberculosis PCR. Tumour markers (AFP, CA125, CA15-3, and CA19-9) were negative. Pericardial fluid was also positive for methicillin-susceptible *S. aureus*.

Empirical antibiotics were initiated with intravenous ceftriaxone and ciprofloxacin.

**Figure 1:**
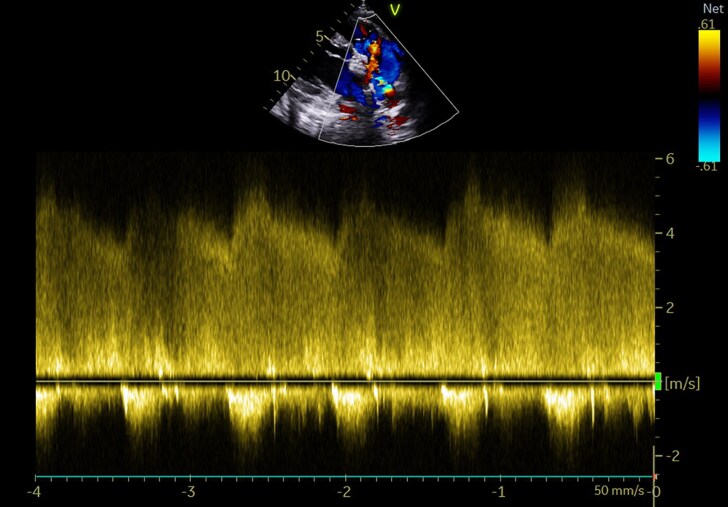
Continuous Doppler echocardiography showing the flow through the patent ductus arteriosus. A continuous flow with a systolic peak of 98 mmHg.

**Figure 2:**
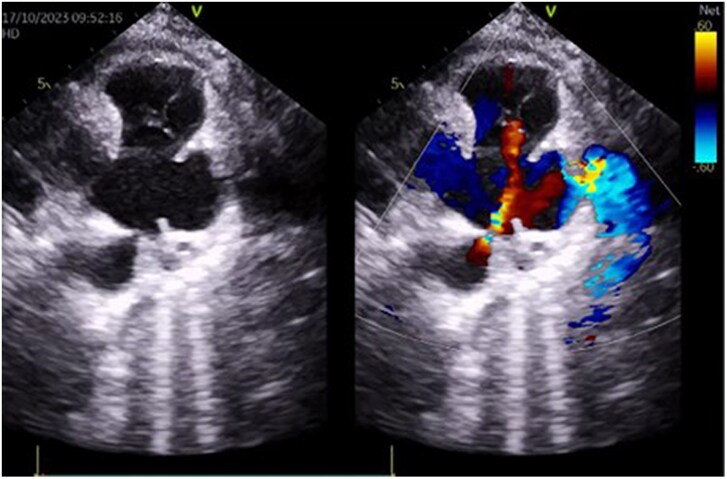
Transthoracic echocardiogram showing a patent ductus arteriosus with a ruptured large aneurysm on the trunk of the pulmonary artery.

The echocardiographic check carried out after the pericardiocentesis revealed the presence of a PDA of 3 mm with left-to-right shunt, no left-sided volume overload, or pulmonary hypertension. The peak gradient through the PDA was 95 mmHg (*Figure 1*). It was associated with an aneurysm of the trunk of the PA measuring 50 × 40 mm ruptured in the pericardium (*Figure 2*), a complementary scan was requested.

**Figure 3:**
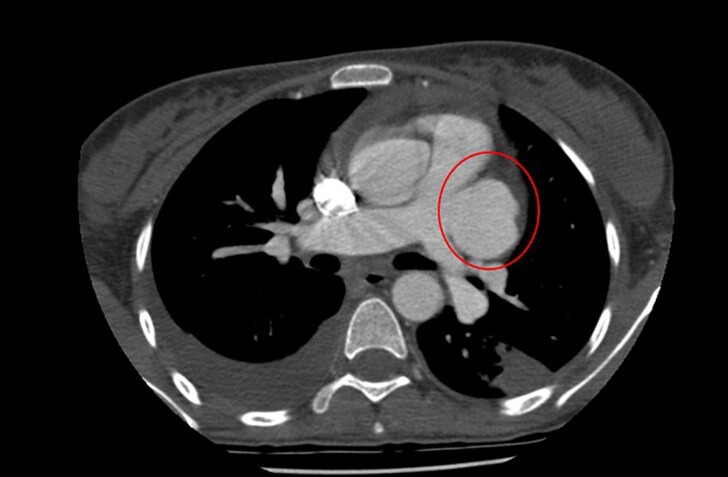
CT scan showing aneurysms on the trunk of the pulmonary artery.

**Figure 4:**
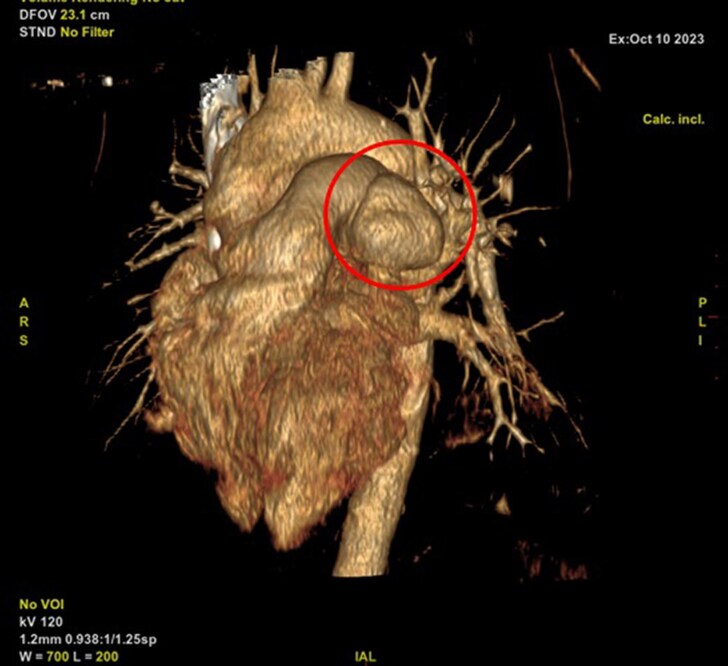
3D reconstruction of a CT scan showing aneurysms on the trunk of the pulmonary artery.

The CT scan revealed: A large aneurysm on the left border of the PA trunk, measuring 58 × 45 mm, with contrast extravasation into the pericardial space, with multiple aneurysms of the right PA, with sizes of 6 × 5 mm and 9 × 6 mm and presence of PDA (*Figures 3* and *4*). There was not any septic pulmonary embolism.

The patient was diagnosed with IE on the PDA, complicated by multiple mycotic aneurysms, with the largest being a partially ruptured aneurysm of the PA trunk.

A complete physical examination and a body scan did not find the point of entry of the infection. In addition, there was not any other location of mycotic aneurysms.

## Management and outcome

**Figure 5:**
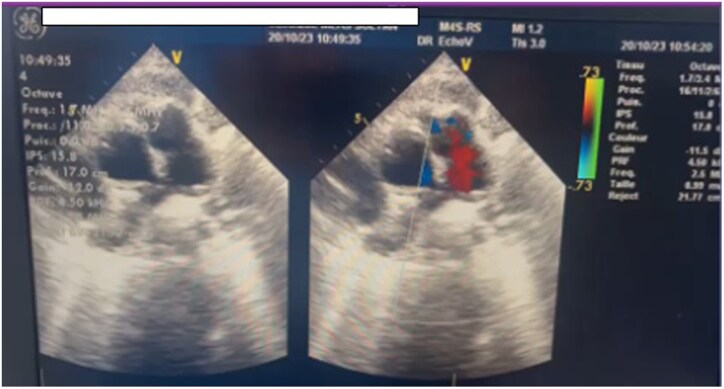
Postoperative transthoracic echocardiography showing no residual pulmonary artery aneurysm.

Emergency surgery was performed, including resection of the aneurysm of the PA trunk, ligation, and sectioning of the PDA, and pericardial drainage. Postoperative TTE on Day 1 revealed no residual PA trunk aneurysm and no pericardial effusion (*Figure 5*), and the patient was hemodynamically stable.

The patient received intravenous bi-antibiotic therapy with 2 weeks of gentamicin and 4 weeks of moxifloxacin. Over the course of treatment, her clinical and biological parameters improved significantly: the inflammatory anaemia improved to 9.7 g/dL, white blood cell count dropped to 13 990/mm³, C-reactive protein level dropped to 6 mg/L, procalcitonin level <0.05 ng/mL, and serum creatinin level of 7.1 mg/L.

Transthoracic echocardiography was repeated at 1-month follow-up revealing no PA aneurysm and no pericardial effusion.

The patient was discharged in good condition. She was followed up at 2 months, 3 months, and then 6 months after discharge with normalizing laboratory results and no recurrence of symptoms.

## Discussion

Although rare, when IE occurs on a PDA, it carries a high risk of severe complications, including the development of mycotic aneurysms.^2^ Mycotic aneurysms are infectious dilations of arteries that can rupture, leading to catastrophic consequences such as haemoptysis, haemothorax, or cardiac tamponade, as observed in this case.

In adults, PDA can often go undiagnosed until complications arise. In this patient, the PDA was complicated by IE caused by *S. aureus*, a common pathogen in IE, known for its aggressive course and high propensity to cause metastatic infections and embolic phenomena.^3–5^ The association between *S. aureus* bacteraemia and the development of mycotic aneurysms is well-documented in the literature, with this pathogen being responsible for the majority of cases.^6^

Mycotic aneurysms are rare in the context of IE but are particularly dangerous. They result from the infection spreading to the arterial wall, causing destruction and subsequent dilation of the vessel.^7^

The rupture of a PA aneurysm into the pericardial space, as seen in this case, is exceedingly rare. It poses immediate life-threatening consequences, necessitating urgent intervention. The mortality rate of ruptured mycotic aneurysms is high, particularly when they occur in large arteries like the PA, due to the potential for rapid haemodynamic collapse.^8^

The diagnosis of mycotic aneurysms in the setting of IE requires a high index of suspicion. CT angiography is the gold standard for diagnosing mycotic aneurysms, as it allows for detailed visualization of the vasculature and any extravasation of contrast, indicative of rupture.^9^

The management of IE complicated by mycotic aneurysms is challenging and requires a multidisciplinary approach involving cardiologists, cardiothoracic surgeons, and infectious disease specialists. In cases where large aneurysms are present or at risk of rupture, surgical intervention is usually necessary. The decision to proceed with surgery is influenced by factors such as the size of the aneurysm, evidence of rupture or impending rupture, and the patient’s overall stability.^10^ The role of antibiotics in managing IE is well-established. ESC guidelines recommend (flu)cloxacillin or cefazolin for 4–6 weeks in methicillin-susceptible *S. aureus* IE.^11^ In this case, the patient was treated with a combination of moxifloxacin and gentamicin, which are effective against *S. aureus*.^12^ Prolonged intravenous antibiotic therapy for at least 4–6 weeks is recommended to ensure complete eradication of the infection, particularly in cases complicated by mycotic aneurysms.^13^

The prognosis of patients with IE complicated by mycotic aneurysms depends on early diagnosis, the extent of the aneurysms, and the timeliness of surgical intervention. According to a study by Nazir *et al*., the mortality rate for ruptured mycotic aneurysms can be as high as 40%, particularly when associated with large vessels like the PA. Early surgery, as was performed in this case, is associated with a significant reduction in mortality and better long-term outcomes.^14^

In a review of cases of mycotic aneurysms associated with IE, Yun *et al*. found that *S. aureus* was the most common pathogen and that surgical intervention, when combined with targeted antibiotic therapy, significantly improved survival rates. However, delays in treatment or failure to recognize the aneurysm early are associated with a poor prognosis.^15^

## Conclusion

This case highlights the rare but severe complication of mycotic aneurysms in the context of IE on a PDA. It underscores the importance of early recognition and intervention. The successful management of this patient through surgical resection of the aneurysm, PDA ligation, and prolonged antibiotic therapy demonstrates the efficacy of a multidisciplinary approach.

## Data Availability

No new data were generated or analysed in support of this article.

## Lead author biography

Professor Meryem Haboub Assistant professor of cardiology in the Faculty of Medicine, Hassan II University, Casablanca, Morocco.
